# Challenges faced when masking a single discoloured tooth - Part 2: indirect restoration procedures

**DOI:** 10.1038/s41415-025-8385-0

**Published:** 2025-07-11

**Authors:** May Aljanahi, Argwan Alhussin, Haitham Elbishari

**Affiliations:** 41415376128001 Hamdan Bin Mohammed College of Dental Medicine (HBMCDM), Mohammed Bin Rashid University of Medicine and Health Sciences (MBRU), Dubai Health, United Arab Emirates; School of Medicine, Dentistry and Biomedical Sciences, Queen´s University Belfast, Belfast, UK; Centre for Public Health, School of Medicine, Dentistry and Biomedical Sciences, Queen´s University Belfast, Belfast, UK; 41415376128002 Hamdan Bin Mohammed College of Dental Medicine (HBMCDM), Mohammed Bin Rashid University of Medicine and Health Sciences (MBRU), Dubai Health, United Arab Emirates; 41415376128003 Hamdan Bin Mohammed College of Dental Medicine (HBMCDM), Mohammed Bin Rashid University of Medicine and Health Sciences (MBRU), Dubai Health, United Arab Emirates; School of Medicine, Dentistry and Biomedical Sciences, Queen´s University Belfast, Belfast, UK; The Faculty of Biology, Medicine and Health, School of Medical Science, Division of Dentistry, University of Manchester, Manchester, England, UK

## Abstract

When conservative approaches are unsuccessful in addressing a single discoloured tooth, prosthetic management becomes essential. This often involves the application of indirect restorations, which provide both functional and aesthetic solutions. These restorations offer durability and improved outcomes, particularly when conservative treatments are inadequate. The integration of prosthetic techniques ensures optimal patient satisfaction and long-term success. This review is the second of a two-article series that will broadly discuss the prosthetic management and challenges faced when masking a single discoloured tooth, due to dark substrates of the crown and the root, and will examine various approaches encompassing veneers, composite veneers and crowns, and the types of cement and its effects. This review aims to highlight the challenges/variables faced when masking discoloured teeth and appraise possible prosthodontics techniques to address this.

## Key points

- Discusses management using indirect restorative techniques, indications and durability.
- Emphasises the importance of cement shade and thickness on masking a discoloured tooth.
- Highlights the challenges faced when masking a single discoloured tooth.

## Indirect restorations

**Fig. 1:**
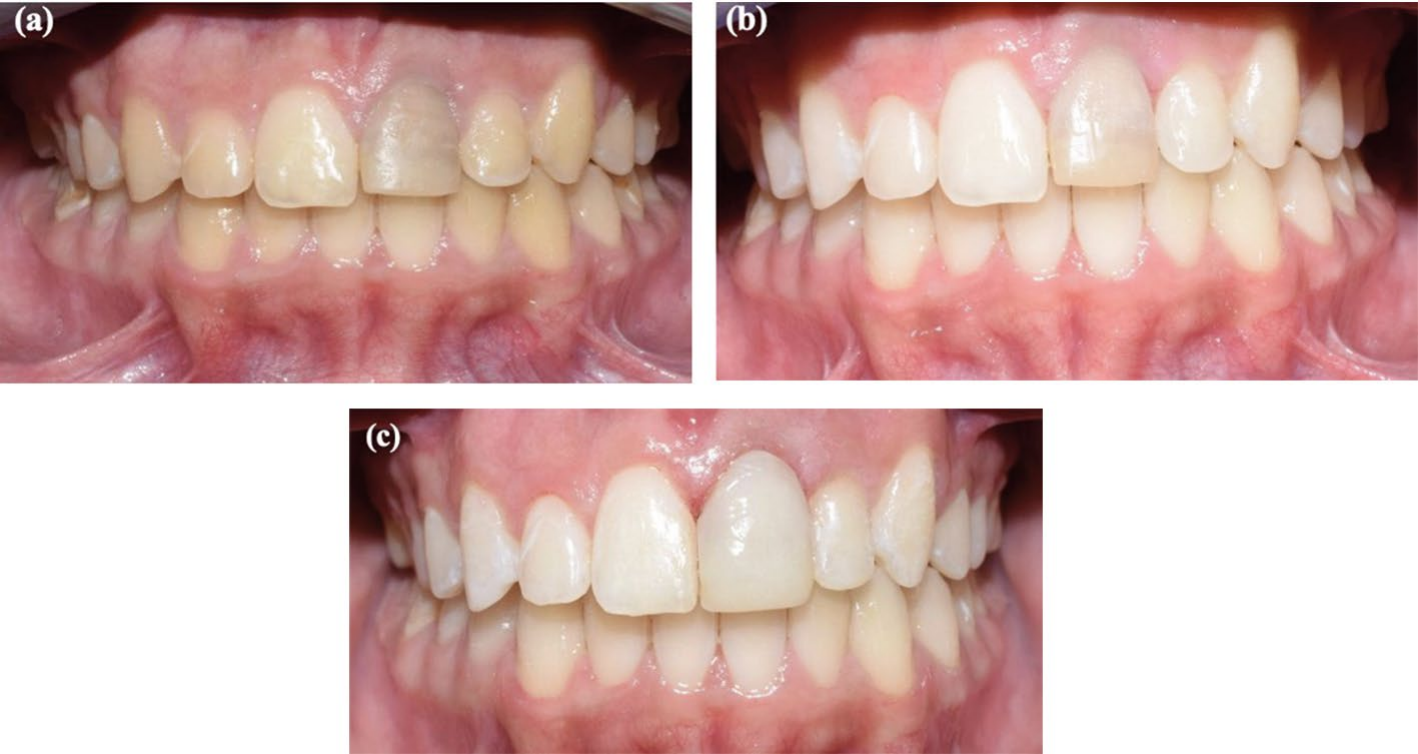
a) A pre-operative photo showing an endodontically-treated tooth discoloration. b) The tooth was managed using inside-outside bleaching using 10-15% carbamide peroxide in a bleaching tray. c) The tooth did not meet the expectations of the patient after the bleaching and was managed by direct composite build-up as a final restoration

Masking a single discoloured tooth remains one of the most demanding aesthetic challenges in restorative dentistry. Factors such as the depth of discoloration, underlying tooth structure, preparation design and material selection all influence the final outcome, particularly when indirect restorations are considered.^1^^,^^2^^,^^3^ When determining the choice between direct or indirect restorative techniques, several factors are considered, including cost, fabrication time, extent of tooth preparation required to mask discoloration, reparability, durability and the patient's expectations.^4^ Indirect restorations, such as indirect composite veneers, ceramic veneers and crowns, are the choice of treatments. The choice of management depends on the type and extent of the discoloration and tooth vitality, and are considered long-term solutions. Figure 1 demonstrates the application of direct composite build up to conceal discoloration despite bleaching being done. Success rates for crowns have been shown to range from 89% at one year to 79% at five years, while veneers are 92% at one year and 80% after five years.^5^ This suggests that while indirect techniques may offer a durable option, they still exhibit challenges, particularly in cases of parafunctional habits or complex restorations.^5^

### Veneers

Veneers can be used to mask and create an illusion, but crowns are more beneficial in attempting to mask moderate to severe discoloration.^6^ More tooth structure removal is removed in crowns, as opposed to 0.5-1 mm for veneers, as well as the type of reduction being done palatally, mesially, distally, incisally and buccally, rather than just labially and incisally.^6^

Composite veneers can be used and are beneficial in young patients (under 16 years of age).^7^ A single discoloured tooth can be masked using composite veneer as it is a reliable and accurate method to camouflage discoloration. Fahl *et al*.^8^ outlined the advantage of tooth shade, shape and anatomy being controlled by the operator for the overall success of the restoration. Other advantages were the high colour match and accuracy, finishing and polishing of the restoration, and margin adaptation.^8^ To overcome the challenges faced when treating with direct composite veneers, indirect composite veneers were introduced, offering improved physical properties, in particular, enhanced wear resistance, hardness, colour stability and biocompatibility.^9^ These improvements result from processing the restorations in a lab or chairside.

Furthermore, a direct-indirect composite veneer technique encompasses the advantages of both approaches and can be completed in one appointment.^10^ The veneer is placed directly onto the tooth, removed, heat-treated, polished and then bonded, ensuring improved physical durability, aesthetic quality and superior marginal adaptation.^10^^,^^11^ The marginal gaps caused by resin polymerisation shrinkage are minimised due to the precise fit of the veneer and a thin film of luting cement.^12^ In cases of both indirect and direct-indirect composite veneers, tooth preparation is necessary. This typically involves a depth of 0.5 mm and a chamfer line within the enamel to ensure a secure seal. In cases where lengthening of the incisal edge is required, the preparation should extend at least 2 mm over the edge to ensure proper integration and durability.^8^

Porcelain laminate veneers have been used to augment and mask discoloration very successfully. Several studies have been done to evaluate the masking potential of all ceramic veneers. Farhan *et al*.^13^ reported the layering technique, where a total number of 40 veneers with various shades of discs was replicated from A1-A4, and the use of bi-laminate and tri-laminate veneers cemented on tooth-coloured ceramic discs. Also, different thicknesses of enamel layers were used and a spectrophotometer was used to measure the change in colour. The results showed that the tri-laminate veneer of 0.8 mm had the best potential to mask a darker discoloured tooth. However, the limitations of this study were that the testing material was a disc similar to other studies, which does not replicate ideal tooth properties of enamel and dentine, and therefore make it difficult to visualise clinically, as well as the use of a single type of ceramic.

Previous studies have been investigating various all-ceramic materials, such as Procera (Nobel Biocare AB), Empress 2 (Ivoclar Vivadent) and Vitadur Alpha (VITA Zahnfabrik), by fabricating disks of shade A2 ceramic to assess the masking potential using a colorimeter.^14^ Chu *et al*.^14^ concluded that Vitadur Alpha had the least potential to mask discoloration as a veneer material over a discoloured tooth. Empress 2 and Procera were able to mask the black discoloration to a certain extent, limiting its use to mild discoloration cases where a yellow or light grey discoloration may be present. The drawback of this study was the comparing of ceramic veneer to crowns, but usually more tooth preparation is required for crowns. The second limitation was that the shade A2 block was used; if a darker block was used, it would have had a higher probability to mask the discoloration. Veneers can be the treatment of choice with mild discoloration, but when teeth are heavily restored and present with dark posts and discoloration, crowns are outlined as the treatment of choice.^15^

The success of veneers depends on case selection, preparation design, material of fabrication and cementation technique, as well as the patient's oral hygiene status and compliance.^16^ According to Nazar *et al.*,^17^ the overall success rate of indirect composite veneers was 83.3%, which was consistent with other studies as well. Additional research concluded restorations on vital teeth resulted in success and survival rates of 86.8% and 95.3%, respectively, while endodontically treated teeth showed success and survival rates of 82.6% and 87.5%, respectively.^16^^,^^18^ Moreover, a randomised controlled trial evaluated the short-term survival rate of composite and ceramic veneers and reported failures in the form of fracture and debonding, and concluded no significance in the survival rates between ceramic and composite veneers.^19^

The success and survival rates of ceramic veneers has been extensively reported in the literature. Aslan *et al*.^20^ reported a 97.4% survival rate after ten years in a no-prep porcelain laminate veneer and complications occurred in 1.64% of the restorations. They concluded that following a strict protocol for placing veneers by experienced dentists resulted in good results. Mihali *et al*.^21^ demonstrated an overall survival rate of 91.7% for up to seven years of function and a failure rate of 8.23%. Their results have shown that the use of feldspathic ceramic veneers using minimally invasive preparation methods achieved a high success rate. Moreover, De Angelis *et al*.^22^ reported a survival rate of 97.4% and a success rate of 91% in a mean observation period of 43 months. The achieved outcome confirmed that a prepless approach resulted in high survival and success rates.

Fracture was the most prevalent failure for veneers, as the restoration is thin and more liable to fracture.^10^ Dentists need to examine occlusion carefully before initiating treatment and after cementation of the veneers, especially in cases of present parafunctional habits, such as bruxism (an unfavorable occlusion which is the leading cause of fractures). Debonding is considered the most common failure after fracture primarily due to lack of mechanical retention from tooth preparation for the veneers. A smaller pre-existing restoration on the tooth to be veneered helps preserve the enamel layer thereby reducing debonding failure, as the loss of enamel further promotes the process of debonding.^10^^,^^23^

### Indirect full coverage

Crowns are extra coronal restorations which can be fabricated from a variety of different materials, such as metal, metal fused to porcelain, or porcelain. Full-coverage crowns are typically indicated in cases of endodontically treated teeth that are heavily restored teeth with posts.^6^ However, patients should be informed about the more extensive loss of tooth structure, higher costs, potential risk and complications, and future repairs and replacement over time.^24^

To mask discoloration, translucent crowns, such as lithium disilicate or leucite-based ceramics, or opaque crowns, such as metal ceramics or zirconia crowns, are used. The advantage of metal-ceramic crowns is that less tooth structure is removed: 1.5 mm in comparison to 2 mm of all-ceramic crowns.^25^ However, a disadvantage is the metal may be visible at the margin. According to Goodacre *et al*.,^26^ axial and occlusal reductions for all-metal crowns should be at least 0.5 mm deep and 1.0 mm deep, respectively. Furthermore, 0.3 mm chamfer lines were determined as the most appropriate for all-metal crowns.^26^ In cases of metal-ceramic crowns, facial or axial reductions in excess of 1 mm can jeopardise the integrity of the tooth structure external to the pulp, whereas 2.0 mm of occlusal reduction is achievable even on a young tooth. For all-ceramic crowns, it is not necessary to exceed 1 mm of axial reduction, and 2 mm incisal-occlusal reduction is recommended for all-ceramic crowns.^26^ In addition, line angles should be rounded, the tooth preparations should be smooth as it enhances the fit of the restorations, and when tooth conditions and aesthetics allow, the finish line should be supragingival.^26^

Clinical examination and judgement outline the choice of material which should be used, as well as the technique and amount of tooth preparation which should be carried out in reference to masking the discoloration of a tooth.

Ceramics have been introduced in dentistry primarily for aesthetics. Ceramics can be further classified into glass ceramics, glass-infiltrated ceramics and oxide ceramics, with each subdivided into different categories, outlined in Table 1.^27^ Furthermore, the type of ceramic will be influenced by its indications, as well as the substructure. The substructure of a ceramic crown will be the tooth and when the tooth presents with a dark discoloration, an opaque ceramic restoration is indicated.^28^

**Table 1:** Classification of dental ceramics

**Glass ceramic**	**Glass-infiltrated ceramic**	**Oxide ceramic**
Amorphous/crystalline sintering temperature <1,000 °C	Porous structure	Polycrystalline sintering temperature >1,350 °C
Feldspathic leucite	In-Ceram alumina	Aluminium oxide
Lithium disilicate	In-Ceram zirconia	Zirconium dioxide

Lithium disilicate is also referred to as Emax (Ivoclar Vivadent) and is part of the synthetic glass matrix ceramics. Its advantageous properties, such as its etching ability, excellent aesthetics and hardness, increases its use in dentistry today. Lithium disilicate crowns are made from a translucent shade of ceramic which allows them to present with the best masking potential when masking mild discoloration. Moreover, Niu *et al*.^29^ concluded an opaque cement can also be used in order to mask discoloration, for instance, when lithium disilicate is indicated, a white opaque cement is more effective in masking the dark colour of silver-palladium alloy and improves the shade match of the lithium disilicate restorations.

Severe discoloration of a single tooth can be masked with opaque crowns, such as metal or zirconia crowns. However, zirconia has the disadvantage that it cannot be etched, hence affecting its micromechanical retention and chemical properties. But to overcome this, zirconia can have pre-surface treatment, such a tribochemical silanation, where alumina particles are bombarded onto the ceramic surface.^30^ It can be effectively cemented using a self-adhesive luting agent that contains 10-methacryloyloxydecyl dihydrogen phosphate (MDP) monomer and has been shown to effectively enhance the bond strength between zirconia and resin cement.^31^ A study by Lim *et al.*^32^ concluded the combination of MDP-containing primer and resin cement was the better adhesive choice following the tribochemical silica coating of zirconia, as it demonstrated significantly higher values of shear bond strength.

**Fig. 2:**
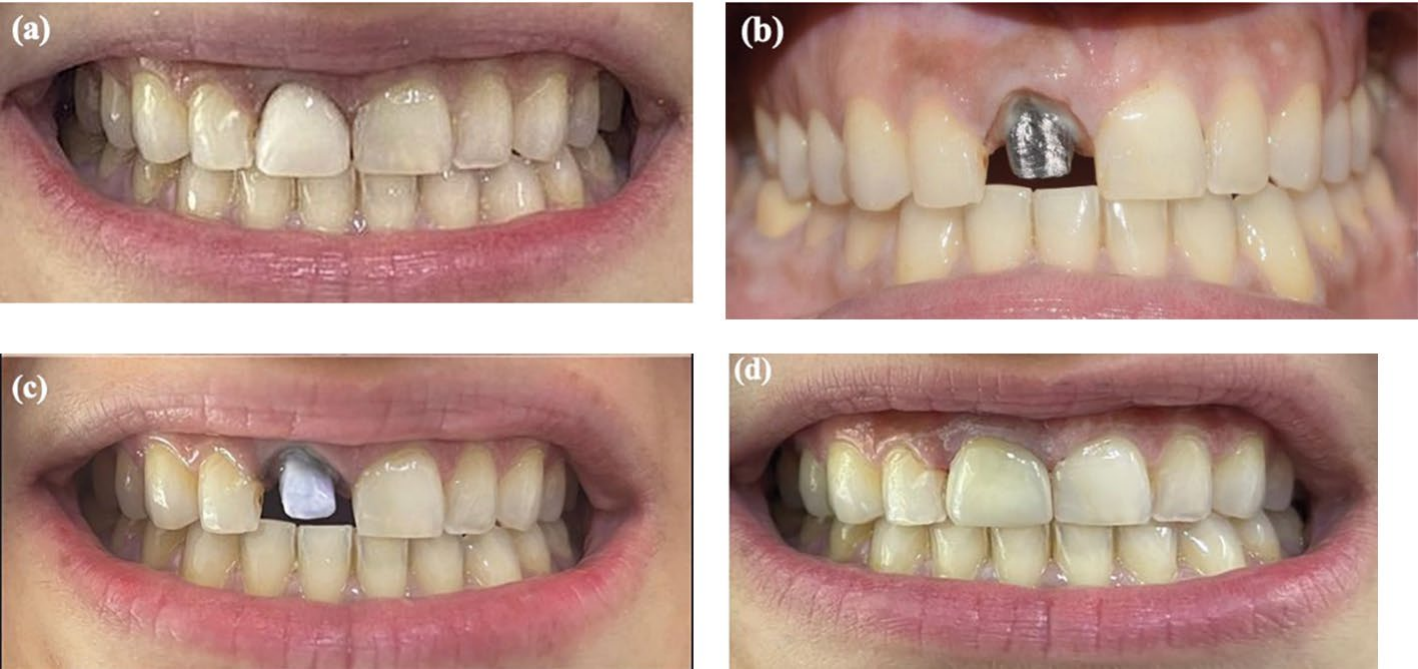
a) A pre-operative photo showing a tooth discoloration. b) The tooth was restored with a dark substrate post and core. c) An opaquer was used to mask the dark discoloration d) The tooth was managed with a lithium disilicate crown to mask the discoloration

In cases of severe discoloration caused by placements of posts, opaquers are used to mask the dark axial wall or to incorporate subtle tones in the restoration.^33^ The opaquer must be chemically compatible with the restoration placed, whether composite or full-coverage crowns, be capable of being light-activated, and must have a consistency that allows for easy placement.^34^ However, it comes with a few limitations. A greyish restoration can be the result of inefficiency of the opaquer to mask the discoloration, or insufficient amount of opaquer applied. Conversely, a matte or very opaque restoration can result from the use of strong opaquers or excessive use.^33^ Figure 2 demonstrates the use of opaquers to mask the dark discoloration as a result of the posts and managed with a lithium disilicate crown.

Choi *et al*.^35^ examined the colour masking potential of zirconia, with and without veneering porcelain. Different substrates of different shades were used, such as white, black, grey and tooth-coloured (VITA shade A3).They measured the change in colour potential by evaluating the initial colour of the disc with a colorimeter, then the colour after two shades of porcelain (A1 and B4) were placed. The results for each group varied and the study concluded that while zirconium oxide coping material alone is able to mask the discoloration, the resulting colour of a restoration can be further modified with the veneering porcelain. A similar study by Tabatabaian *et al*.^36^ fabricated ten zirconia disks by CAD/CAM (computer-aided design/computer-aided manufacturing), each 0.5 mm in thickness and 10 mm in diameter. The disks were divided into four substrates, including a control group (white), light grey, dark grey and black. The results showed that the tested zirconia failed to exhibit high masking potential when masking dark discoloration such as the grey and black substrates.

## Cements

The cementation process of indirect restorations is pivotal in both prosthetic and restorative dentistry. Dental luting cements, used to ensure retention and stabilisation of the restorations, are categorised by their chemical composition and application methods.^37^ Various cements are used in dentistry, each possessing specific properties, such as being temporary versus permanent, dual-cured versus light-cured and resin-based cements versus water-based cements.

**Fig. 3:**
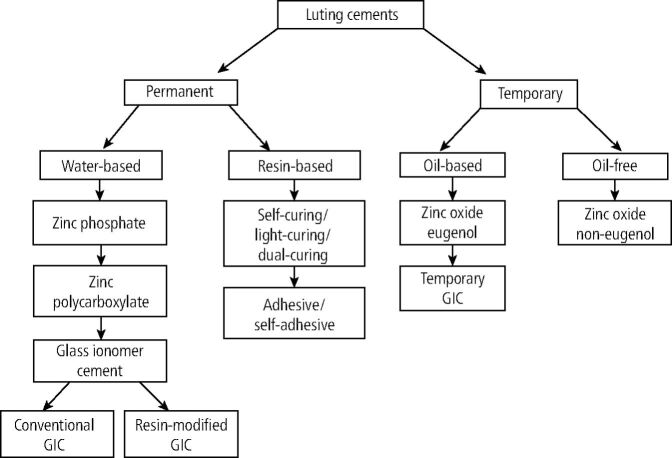
Classification of dental cements

The type, shade and thickness of the resin cement, along with the cement's shade, play a significant role in determining the final colour of the restoration. Figure 3 highlights the various types of luting cements. Water-based cements, like glass ionomer and zinc phosphate, release fluoride, providing additional protective benefits.^37^ On the other hand, resin cements provide maximum strength and are typically used when stronger adhesive bonding is required between the tooth and the restoration.^38^

### Cement shade

The majority of dental cements are opaque in nature, a characteristic that can be beneficial when attempting to conceal a discoloured tooth, as it can camouflage and disguise any discoloration which may be present.^39^ Moreover, this is shown when using Panavia cement, a dual-cured resin cement that can be used when cementing a resin-bonded bridge, since the metal wing can be blocked out by using the opaque cement.^40^ However, when using lithium disilicate, which is translucent itself, applying an opaque cement, especially in the anterior region, may affect aesthetics and be a hindrance.

Cement shade can influence the final restoration, as has been supported in the literature. This becomes a challenge when attempting to mask a discoloured tooth in the anterior aesthetic zone on a patient with a high smile line.^40^ The use of try-in paste can be beneficial at this stage, which highlights the importance of a try-in visit rather than a final cementation after impression taking. Nevertheless, the operator should not solely be dependent on the try-in paste alone, since Mourouzis *et al*.^41^ determined that Variolink try-in pastes do not mimic the exact shade of cements used when cementing IPS Emax CAD restorations.

### Cement thickness

Cement thickness is also an element which contributes to potential masking. Cement thickness is determined by the operator during mixing. Ideally, a low cement thickness is required for better adaptability and seating of the crown.^41^ Vichi *et al.*^42^ explored various cement thickness of 0.1 mm and 0.2 mm and its effect on the ceramic crown. They highlighted the importance of how a dark substructure can negatively influence the crown being placed. The experiment conducted focused on how dark posts have a detrimental outcome on a ceramic crown. The study examined a zirconia, carbon fibre and experimental ceramic post and a Variolink II cement where a spectrophotometer was used to measure the change of colour with four substrates.

The results concluded that the ideal thickness of ceramic which would not be influenced by the cement shade was 2.0 mm. Lowering the ceramic thickness will mean that additional measures need to be considered to mask any underlying discoloration. Lowering the cement thickness to 1.0 mm will not only affect the crown's resistance and retention features but will also fail to mask any discoloration and lead to poor aesthetics. For instance, crowns fabricated with leucite glass ceramic (IPS Empress) will fracture under occlusal loading when the thickness is less than 1.5 mm. All the mechanical properties of the crown, as well as the aesthetic and biological properties at the time of cementation, will affect the outcome as well as the longevity of a restoration.^40^ However, the drawbacks of this study were that one type of ceramic was investigated, the use of only translucent cements of various shades, and the discoloured substrates that were under examination had no metal component to it.

Niu *et al.*^29^ investigated both aspects of shade as well as cement thickness in regard to the colour of machinable lithium disilicate ceramic luted on silver palladium substructures. Here, 1.5 mm ceramic thick blocks were extracted from shade A1 lithium disilicate blocks, which was standard for all specimens. Additionally, five different types of resin cement were taken into consideration, each cement varying in terms of colour and opacities, with ranges from 50-300 micrometres. The study revealed that both cement colour as well as thickness had an effect regarding the coronal lithium disilicate restoration. The optimal cement thickness for colour match varied among cements of different opacities; however, no difference was noted when the cement thickness was above 100 micrometres. The limitation of this study was the use of ceramic with a thickness of 1.5 mm, as many studies, such as Vichi *et al.*,^42^ suggest that a minimum ceramic thickness of 2 mm is required to mask discoloration. Additional limitations include the absence of which bonding agent is being used, which is important since it does not allow the operator to visualise the study in a clinical scenario.

The spectrophotometer is an instrument that measures the amount of light that a sample can absorb.^43^ The Vita EasyShade Compact (Vident) is a small, cordless and portable spectrophotometer that was designed for intra-oral and extra-oral dental shade matching. It gained popularity in recent dental research for its use in shade matching and numerous studies confirming its reliability and precision.^44^ When comparing with subjective visual assessments, spectrophotometers improve accuracy with 33% and provide an objective shade match in 93.3% of patients.^45^ However, Douglas *et al.*^46^ measured the change in colour with a spectroradiometer as it can determine the true colours of translucent specimens and avoid the inaccuracies of edge loss. Nonetheless, spectrophotometers are considered the leading and foremost tool in determining shade, since they present with advantages such as replication of results, accuracy and precision.^29^ However, colour measurements of translucent materials performed with a spectrophotometer can be prone to deviations caused by edge-loss effect. Moreover, the tool is costly and would explain the popularity of manual shade guides being used to assess various hues and shades.^29^^,^^45^

## Challenges faced when masking a discoloured tooth

Many challenges can be faced by clinicians when attempting to diagnose and manage a discoloured tooth. The discoloration complexity scale was developed to aid clinicians in assessing the severity of the discoloration and planning appropriate management approaches. This scale is classified into mild, moderate and severe, as outlined in Table 2.

**Table 2:** Discoloration complexity scale

**Mild**	**Moderate**	**Complex**
Low smile line A vital single discoloured tooth Generalised mild staining No loss of tooth structure Easy to match to adjacent teeth	Average smile line A non-vital single discoloured tooth (grey discoloration) No/minimal loss of tooth structure	High smile line Severe discoloured tooth Discoloured post Loss of tooth structure Difficult to match to adjacent teeth

- Mild cases can be treated conservatively with minimal intervention and vital tooth bleaching
- Moderate cases can be treated by non-vital bleaching or in some instances, direct or indirect composite/ceramic veneers
- Complex cases require extensive treatment of bleaching followed by indirect full coverage.

**Fig. 4:**
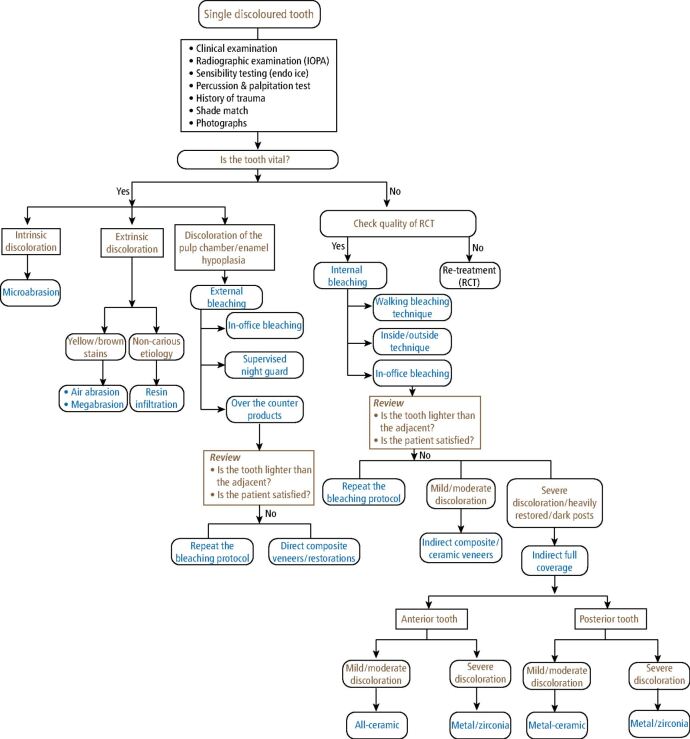
Guidelines for management of a single discoloured tooth

Figure 4 demonstrates the steps involved in the management of a discoloured tooth.

## Conclusion

Many variables are faced when masking a discoloured tooth, as well managing it. Since the underlying discoloration shines and reflects through, it tends to draw and attract the naked eye to it. Several factors have been considered in order to camouflage this, such as the amount of tooth preparation, the choice of material, and the type and thickness of cement, as well as internal, external, or walking bleaching. This final review, second in a two-part series, has provided a comprehensive explanation of the invasive treatment options in cases where minimally invasive procedures are not deemed suitable.

## Data Availability

The data analysed in this review are available from the corresponding author on request.

## References

[1] Romero M F. Esthetic anterior composite resin restorations using a single shade: step-by-step technique. *J Prosthet Dent* 2015; **114:** 9-12. 10.1016/j.prosdent.2015.02.013 25917855

[2] Alothman Y, Bamasoud M S. The success of dental veneers according to preparation design and material type. *Open Access Maced J Med Sci* 2018; **6:** 2402-2408. 10.3889/oamjms.2018.353 PMC6311473 30607201

[3] Coelho-de-Souza F H, Gonçalves D S, Sales M P *et al.* Direct anterior composite veneers in vital and non-vital teeth: a retrospective clinical evaluation. *J Dent* 2015; **43:** 1330-1336. 10.1016/j.jdent.2015.08.011 26318419

[4] Lin J, Bennani V, Aarts J M, Brunton P, Ratnayake J. Factors influencing success rate of ceramic veneers on endodontically treated anterior teeth: a systematic review. *J Prosthet Dent* 2023; DOI: 10.1016/j.prosdent.2023.10.031. 10.1016/j.prosdent.2023.10.031 38030544

[5] Akshay K P U, Krishna P L. *Survivability of Crown and Veneers on ET Anterior Teeth*. Saarbrücken: LAP Lambert Academic Publishing, 2023.

[6] Barber A J, King P A. Management of the single discoloured tooth part 2: restorative options. *Dent Update* 2014; **41:** 194-204. 10.12968/denu.2014.41.3.194 24839707

[7] Wray A, Welbury R. Treatment of intrinsic discoloration in permanent anterior teeth in children and adolescents. *Int J Paediatr Dent* 2001; **11:** 309-315. 10.1046/j.1365-263x.2001.00300.x 11570449

[8] Fahl Júnior N. The direct/indirect composite resin veneers: a case report. *Pract Periodontics Aesthet Dent* 1996; **8:** 627-640. 9242137

[9] Covey D A, Tahaney S R, Davenport J M. Mechanical properties of heat-treated composite resin restorative materials. *J Prosthet Dent* 1992; **68:** 458-461. 10.1016/0022-3913(92)90410-c 1432761

[10] Alghazzawi T F. Clinical survival rate and laboratory failure of dental veneers: a narrative literature review. *J Funct Biomater* 2024; **15:** 131. 10.3390/jfb15050131 PMC11122289 38786642

[11] Lim T W, Tan S K, Li K Y, Burrow M F. Survival and complication rates of resin composite laminate veneers: a systematic review and meta-analysis. *J Evid Based Dent Pract* 2023; **23:** 101911. 10.1016/j.jebdp.2023.101911 38035903

[12] Freire A, Archegas L R P. Porcelain laminate veneer on a highly discoloured tooth: a case report. *J Can Dent Assoc* 2010; **76:** 126. 20943031

[13] Farhan D, Sukumar S, von Stein-Lausnitz A, Aarabi G, Alawneh A, Reissmann D R. Masking ability of bi- and tri- laminate all-ceramic veneers on tooth-colored ceramic discs. *J Esthet Restor Dent* 2014; **26:** 232-239. 10.1111/jerd.12099 24980698

[14] Chu F C S, Chow T W, Chai J. Contrast ratios and masking ability of three types of ceramic veneers. *J Prosthet Dent* 2007; **98:** 359-364. 10.1016/S0022-3913(07)60120-6 18021824

[15] Antonson S A, Anusavice K J. Contrast ratio of veneering and core ceramics as a function of thickness. *Int J Prosthodont* 2001; **14:** 316-320. 11508085

[16] Gresnigt M M M, Kalk W, Ozcan M. Randomized controlled split-mouth clinical trial of direct laminate veneers with two micro-hybrid resin composites. *J Dent* 2012; **40:** 766-775. 10.1016/j.jdent.2012.05.010 22664565

[17] Nazar A, Bader Munir M, Rafiq A, Khalid S, Hassan H. Success of veneers with indirect resin composite. *Pak J Med Health Sci* 2021; **15:** 3619-3622.

[18] Li X, Wu B, Cheng X, Li Y, Xie X, Deng F. Esthetic evaluation of implant-supported single crowns: the implant restoration esthetic index and patient perception. *J Prosthodont* 2019; DOI: 10.1111/jopr.12659. 10.1111/jopr.12659 29148207

[19] Gresnigt M M, Kalk W, Ozcan M. Randomized clinical trial of indirect resin composite and ceramic veneers: up to 3-year follow-up. *J Adhes Dent* 2013; **15:** 181-190. 10.3290/j.jad.a28883 23534025

[20] Aslan Y U, Uludamar A, Özkan Y. Retrospective analysis of lithium disilicate laminate veneers applied by experienced dentists: 10-year results. *Int J Prosthodont* 2019; **32:** 471-474. 10.11607/ijp.6234 31664262

[21] Mihali S G, Lolos D, Popa G, Tudor A, Bratu D C. Retrospective long-term clinical outcome of feldspathic ceramic veneers. *Materials (Basel)* 2022; **15:** 2150. 10.3390/ma15062150 PMC8954582 35329602

[22] De Angelis F, D'Arcangelo C, Angelozzi R, Vadini M. Retrospective clinical evaluation of a no-prep porcelain veneer protocol. *J Prosthet Dent* 2023; **129:** 40-48. 10.1016/j.prosdent.2021.04.016 34059296

[23] Sadighpour L, Geramipanah F, Rasaei V, Kharazi Fard M J. Fracture resistance of ceramic laminate veneers bonded to teeth with class V composite fillings after cyclic loading. *Int J Dent* 2018; **2018:** 1456745. 10.1155/2018/1456745 PMC5932446 29849632

[24] Kahler B. Present status and future directions - managing discoloured teeth. *Int Endod J* 2022; **55:** 922-950. 10.1111/iej.13711 PMC9790475 35188275

[25] Shillingburg H T, Sather D A, Wilson E L *et al.**Fundamentals of Fixed Prosthodontics*. Chicago: Quintessence Publishing Company, 2012.

[26] Goodacre C J, Campagni W V, Aquilino S A. Tooth preparations for complete crowns: an art form based on scientific principles. *J Prosthet Dent* 2001; **85:** 363-376. 10.1067/mpr.2001.114685 11319534

[27] Stawarczyk B, Keul C, Eichberger M, Figge D, Edelhoff D, Lümkemann N. Three generations of zirconia: from veneered to monolithic, part I. *Quintessence Int* 2017; **48:** 369-380. 10.3290/j.qi.a38057 28396886

[28] Gracis S, Thompson V, Ferencz J, Silva N, Bonfante E. A new classification system for all-ceramic and ceramic-like restorative materials. *Int J Prosthodont* 2016; **28:** 227-235. 10.11607/ijp.4244 25965634

[29] Niu E, Agustin M, Douglas R D. Color match of machinable lithium disilicate ceramics: effects of cement color and thickness. *J Prosthet Dent* 2014; **111:** 42-50. 10.1016/j.prosdent.2013.09.005 24210729

[30] Zakir M, Ashraf U, Tian T *et al*. The role of silane coupling agents and universal primers in durable adhesion to dental restorative materials - a review. *Curr Oral Health Rep* 2016; **3:** 244-253.

[31] Carrabba M, Nagasawa Y, Parrini S, Doldo T, Wood D, Ferrari M. Zirconia translucency and cement systems as factors influencing the zirconia-titanium and zirconia-zirconia shear bond strength. *Int J Oral Maxillofac Implants* 2019; **34:** 1053-1058. 10.11607/jomi.7212 31528861

[32] Lim M-J, Yu M-K, Lee K-W. The effect of continuous application of MDP-containing primer and luting resin cement on bond strength to tribochemical silica-coated Y-TZP. *Restor Dent Endod* 2018; DOI: 10.5395/rde.2018.43.e19. 10.5395/rde.2018.43.e19 PMC5952057 29765899

[33] Felippe L A, Monteiro S Jr, Baratieri L N, Caldeira de Andrada M A, Ritter A V. Using opaquers under direct composite resin veneers: an illustrated review of the technique. *J Esthet Restor Dent* 2003; **15:** 327-337. 10.1111/j.1708-8240.2003.tb00306.x 14982659

[34] Felippe L A, Baratieri L N. Direct resin composite veneers: masking the dark prepared enamel surface. *Quintessence Int* 2000; **31:** 557-562. 11203977

[35] Choi Y-J, Razzoog M E. Masking ability of zirconia with and without veneering porcelain. *J Prosthodont* 2013; **22:** 98-104. 10.1111/j.1532-849X.2012.00915.x 23387963

[36] Tabatabaian F, Javadi Sharif M, Massoumi F, Namdari M. The color masking ability of a zirconia ceramic on the substrates with different values. *J Dent Res Dent Clin Dent Prospects* 2017; **11:** 7-13. 10.15171/joddd.2017.002 PMC5390131 28413589

[37] Heboyan A, Vardanyan A, Karobari M I *et al.* Dental luting cements: an updated comprehensive review. *Molecules* 2023; **28:** 1619. 10.3390/molecules28041619 PMC9961919 36838607

[38] Dos Santos V H, Griza S, de Moraes R R, Faria-E-Silva A L. Bond strength of self-adhesive resin cements to composite submitted to different surface pretreatments. *Restor Dent Endod* 2014; **39:** 12-16. 10.5395/rde.2014.39.1.12 PMC3916500 24516824

[39] Raigrodski A J. Contemporary materials and technologies for all-ceramic fixed partial dentures: a review of the literature. *J Prosthet Dent* 2004; **92:** 557-562. 10.1016/j.prosdent.2004.09.015 15583562

[40] Gulati J S, Tabiat-Pour S, Watkins S, Banerjee A. Resin-bonded bridges - the problem or the solution? Part 2: practical techniques. *Dent Update* 2016; **43:** 608-616. 10.12968/denu.2016.43.7.608 29148671

[41] Mourouzis P, Koulaouzidou E, Palaghias G, Helvatjoglu-Antoniades M. Color match of luting composites and try-in pastes: the impact on the final color of CAD/CAM lithium disilicate restorations. *Int J Esthet Dent* 2018; **13:** 98-109. 29379906

[42] Vichi A, Ferrari M, Davidson C L. Influence of ceramic and cement thickness on the masking of various types of opaque posts. *J Prosthet Dent* 2000; **83:** 412-417. 10.1016/s0022-3913(00)70035-7 10756290

[43] Luo W, Westland S, Ellwood R, Pretty I, Cheung V. Development of a whiteness index for dentistry. *J Dent* 2009; DOI: 10.1016/j.jdent.2009.05.011. 10.1016/j.jdent.2009.05.011 19501446

[44] Khashayar G, Dozic A, Kleverlaan C J, Feilzer A J. Data comparison between two dental spectrophotometers. *Oper Dent* 2012; **37:** 12-20. 10.2341/11-161-C 21942236

[45] Chu S J, Trushkowsky R D, Paravina R D. Dental color matching instruments and systems. Review of clinical and research aspects. *J Dent* 2010; DOI: 10.1016/j.jdent.2010.07.001. 10.1016/j.jdent.2010.07.001 20621154

[46] Douglas R D, Steinhauer T J, Wee A G. Intraoral determination of the tolerance of dentists for perceptibility and acceptability of shade mismatch. *J Prosthet Dent* 2007; **97:** 200-208. 10.1016/j.prosdent.2007.02.012 17499089

